# A Thematic Qualitative Synthesis on the Lived Experiences of Patients With Left Ventricular Assist Devices: A Journey From Vulnerability to Resilience

**DOI:** 10.1111/nicc.70295

**Published:** 2026-01-09

**Authors:** Dimitra Koutsavli, Antonia Kalogianni, Evdokia Misouridou, Eleftheria Brigitta Souvatzi, Fiona Timmins, Stelios Parissopoulos

**Affiliations:** ^1^ Department of Nursing University of West Attica Athens Greece; ^2^ Department of Thoracic and Cardiovascular Surgery “Evangelismos” General Hospital of Athens Athens Greece; ^3^ Department of Electrical and Electronics Engineering University of West Attica Athens Greece; ^4^ School of Nursing and Midwifery University College Dublin Dublin Ireland

**Keywords:** coping strategies, critical care nursing, left ventricular assist device (LVAD), lived experience, qualitative synthesis

## Abstract

**Background:**

Left ventricular assist devices (LVADs) are increasingly used in managing advanced heart failure. While their clinical benefits are well documented, less is known about how patients experience living with such life‐sustaining devices. Understanding these experiences is essential for holistic, person‐centred nursing care.

**Aim:**

To explore the lived experiences of LVAD recipients and examine how they cope with the emotional, psychological and social challenges of life with an LVAD.

**Study Design:**

A systematic search was conducted across Scopus, PubMed and ScienceDirect using predefined terms. The retrieved studies were reviewed and subjected to qualitative synthesis according to Thomas and Harden's methodology. Screening was performed in Covidence, critical appraisal utilised CASP criteria, coding was carried out in ATLAS.ti, and reporting followed ENTREQ guidelines.

**Findings:**

Sixteen qualitative studies published between 2010 and 2024 were reviewed. Four analytical themes were identified: (1) *Loss: The Failing Body, Stigma, and the Others*, (2) *Living with Fear and Uncertainty: And Now What?* (3) *Liminality: Stagnating Present and Ambivalence* and (4) *Moving Forward and Finding New Meaning in Life*. These reflect a psychological and existential journey from vulnerability to resilience, aligning with Lazarus and Folkman's Transactional Model of Stress and Coping.

**Conclusions:**

The synthesis offers novel insights into how LVAD patients cope with complex challenges and highlights underexplored domains such as stigma, identity disruption and meaning‐making.

**Relevance to Clinical Practice:**

Findings underscore the need for holistic, continuous care strategies that support both the technological and psychosocial adaptation of LVAD patients, beginning in the intensive care unit. ICU nurses are pivotal in initiating recovery by providing early psychological support, fostering patient empowerment and preparing individuals and their families for life after critical illness. Addressing emotional instability, identity disruption and fear during ICU admission and early discharge planning can promote resilience and smoother transitions. This review highlights the crucial role of ICU nurses not only in acute care delivery but also in shaping long‐term coping trajectories. Applying the Transactional Model of Stress and Coping offers a novel lens for guiding these interventions across the critical care continuum.

## Impact Statements

- What is known about the topic
  ○ LVAD therapy improves survival in patients with advanced heart failure, but it introduces significant psychosocial challenges.
  ○ Psychological distress, fear of device failure and social isolation are commonly reported but are often under‐addressed in clinical practice.
  ○ Critical care nursing focuses primarily on acute care, with limited attention given to long‐term psychosocial outcomes post‐ICU.
- What this paper adds
  ○ Offers a qualitative synthesis of LVAD patient experiences, revealing a journey from vulnerability to resilience.
  ○ Demonstrates that emotional instability, existential fear and disrupted identity begin during critical illness and must be addressed from the ICU onwards.
  ○ Highlights how ICU nurses can play a key role in psychological support, anticipatory guidance and preparing patients for life with an LVAD.
  ○ Proposes that holistic critical care—addressing spiritual, emotional and social needs—can improve long‐term adaptation and quality of life.
  ○ Recommends strengthening ICU discharge practices and follow‐up strategies to improve continuity of care and psychosocial recovery.

## 1 Introduction

Heart failure (HF) affects approximately 37.7 million people worldwide, with its prevalence steadily increasing [1, 2]. In Europe, the incidence is about 5 per 1000 people annually, and 12% of adults are affected [3]. Advanced HF significantly impairs physical function, increases dependence on others and severely reduces quality of life [4].

Left ventricular assist devices (LVADs) are primarily used for advanced HF, particularly in patients with predominant left ventricular systolic dysfunction. LVADs have become standard therapy, improving survival rates with 1‐year survival exceeding 80% and 2‐year survival above 70% [5]. LVADs are primarily used as a bridge to heart transplantation (BTT) or, in cases where transplantation is not viable, as destination therapy (DT) [6, 7]. These devices enhance health‐related quality of life (HRQoL) by alleviating symptoms and improving both physical and psychological well‐being. The LVAD significantly impacts patient care in critical settings by introducing complex challenges and demands for both patients and healthcare providers. LVADs improve survival and quality of life for patients with end‐stage heart failure but introduce significant challenges in critical care settings. Addressing these challenges through structured training, adequate staffing and improved patient education can enhance care quality and reduce the burden on healthcare providers [8].

## 2 Background/Justification for Study

Despite these benefits, several challenges remain. Barriers to wider LVAD use include the burden of self‐care, limited access to expert care outside implanting centres, persistent device‐related complications and lower heart transplant likelihood for bridge‐to‐transplant patients [5]. Additionally, managing right heart failure post‐LVAD implantation requires careful perioperative and postoperative strategies [9]. Technological advancements, such as fully magnetically levitated pumps, have improved the durability, haemocompatibility and overall outcomes of LVADs, with survival rates now comparable to heart transplantation [10, 11, 12]. However, adverse events continue to challenge patient well‐being, potentially affecting HRQoL [13].

LVAD implantation also introduces new demands for patients and caregivers, ranging from basic daily care to more complex tasks, such as device monitoring, alarm troubleshooting and managing complications. The psychosocial impact, device management complexities and burden of adverse events remain significant concerns [14]. A systematic review by Hahn et al. [14] highlighted improvements in physical and mental HRQoL post‐LVAD, but further research is needed to understand the full scope of benefits and burdens, particularly by incorporating patient feedback to improve safety and satisfaction [15].

While quantitative studies have extensively examined LVAD outcomes, qualitative research exploring patients' lived experiences remains limited, and only a few systematic reviews have addressed this important area [16]. Thematic synthesis of qualitative studies, though rare, provides unique and valuable insights that go beyond clinical outcomes—deepening our understanding of patients' experiences of illness, psychological adaptation, identity and the complex care needs of patients living with LVADs [17].

## 3 Aims and Objectives

This study employs Thomas and Harden's [18] thematic qualitative synthesis methodology to integrate qualitative findings systematically. This approach is especially useful for identifying key factors influencing psychological adaptation and coping, with a focus on individual attitudes and environmental support [19]. By synthesising qualitative data on LVAD recipients' experiences, the study aims to offer a nuanced understanding of the complex interactions between technology, care and the psychological impacts of LVAD therapy, ultimately informing better care strategies and future research [2].

## 4 Design and Methods

This systematic review follows the guidelines outlined in the Enhancing Transparency in Reporting the Synthesis of Qualitative Research (ENTREQ) checklist (Table S1), which aims to improve the consistency, rigour and transparency in reporting the synthesis of qualitative research [20]. The review was conducted in adherence to best practices for systematic qualitative reviews, including a comprehensive search strategy, double‐blind screening and quality assessment of included studies. The review question was formulated according to the PerSPE(C)TiF approach [21] (Table 1).

**TABLE 1:** Review question formulation using PerSPE(C)TiF [21].

	Review question
Perspective	Adult recipients of a left‐ventricular assist device (LVAD) describing their own thoughts, feelings and day‐to‐day realities after implantation
Setting	The full care‐continuum in which LVAD patients live and receive care: From acute hospital environments (ICU, cardiac wards) through rehabilitation to home and community life
Phenomenon/problem	Lived experience of, and coping with, the emotional, psychological, social and practical challenges of depending on an LVAD for survival
Environment	Contemporary advanced‐heart‐failure care characterised by high‐tech, life‐prolonging therapy, complex self‐management demands, risk of complications and a growing emphasis on holistic, person‐centred nursing support
(Optional) Comparison	Not applicable—The synthesis does not compare experiences with another device or therapy
Timing	Across the whole trajectory: Peri‐implantation, early adjustment and long‐term life with an LVAD, recognising that experiences evolve from hospital admission through post‐discharge survivorship
Findings (intended users)	Actionable insights for critical‐care and cardiac nurses, multidisciplinary LVAD teams, service planners and policymakers to design holistic, stigma‐sensitive support that fosters coping, autonomy and quality of life

### 4.1 Search

The electronic databases PubMed, Scopus and Science Direct were systematically searched for literature published until 31 December 2024. Two researchers D.K. and E.B.S. developed and piloted relevant search terms in a preliminary search. The final strategy included a combination of terms related to methodology, population and intervention, adapted to each database's search operators. The search was limited to articles published in English from 2010 to 2024. The search focused on three concepts: (a) ‘LVAD’, (b) ‘patients’ and (c) ‘qualitative’ (Table S1). For example, the search strategy at PubMed is presented below:

((‘heart‐Assist Devices’[MeSH Terms] OR ‘left ventricular assist device*’[Text Word] OR ‘LVAD’[Text Word] OR ‘mechanical circulatory support*’[Text Word] OR ‘heart assist device*’[Text Word]) AND (‘patients/education’[MeSH Terms] OR ‘patients/psychology’[MeSH Terms] OR ‘patients/therapy’[MeSH Terms] OR ‘Caregiver Burden’[MeSH Terms] OR (‘heart failure/nursing’[MeSH Terms] OR ‘heart failure/surgery’[MeSH Terms]) OR ‘coordinator*’[Text Word]) AND (‘qualitative research*’[MeSH Terms] OR ‘Life Change Events’[MeSH Terms] OR ‘Personal Narratives as Topic’[MeSH Terms] OR ‘Nursing Methodology Research’[MeSH Terms] OR ‘experience*’[Text Word] OR ‘phenomenolog*’[Text Word])) AND (2010:2022[pdat]).

The search yielded 529 records, with 24 duplicates identified across databases. Additionally, one article was found manually through an internet search.

### 4.2 Screening Process and Inclusion Criteria

**FIGURE 1:**
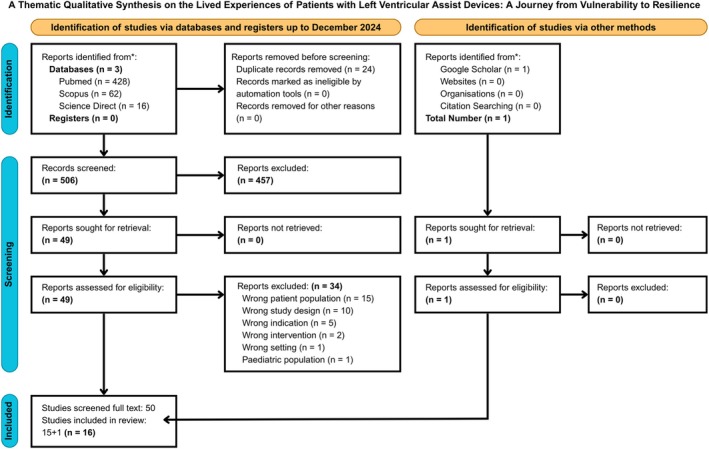
PRISMA flow diagram depicting the study selection process for thematic synthesis of LVAD patient experiences. The chart outlines the systematic review process, including database searches, screening, eligibility assessment and final inclusion. A total of 507 records were identified across multiple databases and other sources, with 24 duplicates removed. Following title and abstract screening, 50 full‐text articles were assessed for eligibility, resulting in 16 studies included in the final thematic synthesis. Reasons for exclusion are detailed for transparency and methodological rigour.

All references were downloaded and imported into the reference management software Covidence (https://www.covidence.org/). After discarding duplicates, the remaining references underwent a two‐stage screening process conducted independently by three reviewers S.P., D.K. and E.B.S. The first stage involved manual, double‐blind screening of abstracts and titles. As a result, 457 records were excluded due to a different population, study design or setting. In the second stage, a full‐text review of 50 studies was conducted independently by three authors (Figure 1).

Studies were eligible for inclusion in this synthesis if they met the following criteria:

- Population: Adult patients living with Left Ventricular Assist Devices (LVADs).
- Language: Published in English.
- Publication Date: Between 2010 and 2024.
- Type of publication: Peer‐reviewed primary research articles.
- Study design: Qualitative studies using qualitative methodologies and reporting on patients' lived experiences.

The decision to focus exclusively on LVAD recipients and their caregivers was based on both practical and methodological considerations. LVADs are predominantly used in adult populations with advanced heart failure and therefore represent the most extensively studied group in the existing literature. In contrast, qualitative evidence related to RVADs (Right Ventricular Assist Devices), BiVADs (Biventricular Assist Devices) and paediatric populations is limited. Including these groups would have introduced substantial clinical heterogeneity, potentially undermining the phenomenological focus on lived experience and complicating the ability to draw meaningful conclusions. Moreover, the clinical trajectories, risks and decision‐making processes in temporary mechanical support (e.g., RVADs, BiVADs) or paediatric contexts differ significantly and warrant separate investigation. The authors also noted that studies involving RVAD or BiVAD patients typically reflect different clinical profiles and indications, further justifying their exclusion. While the study samples include both bridge‐to‐transplant (BTT) and destination therapy (DT) LVAD recipients, thematic differences between these subgroups remain under exploration. Additionally, many of the studies reviewed did not clearly distinguish between BTT and DT cohorts, limiting the authors' ability to analyse them separately.

In cases of disagreement, studies were discussed until consensus was reached. The inclusion criteria for this systematic review were qualitative studies with adult participants. Studies were excluded if they focused on caregivers, used mixed methods or were published before 2010. Ultimately, 16 qualitative studies were included in the review (Table 2) [22, 23, 24, 25, 26, 27, 28, 29, 30, 31, 32, 33, 34, 35, 36, 37].

**TABLE 2:** The studies included in the review.

Authors (publication year)	Title of the study
1. Bechthold et al. (2024) [22]	“When I do have some time, rather than spend it polishing silver, I want to spend it with my grandkids”: A qualitative exploration of patient values following left ventricular assist device implantation
2. Inyom et al. (2022) [23]	Lived experiences of patients implanted with left ventricular assist devices
3. Treß et al. (2022) [24]	Balancing normalcy and safety: health‐related needs in patients with a ventricular assist device within their home environment
4. Levelink et al. (2022) [25]	Psychological burden and coping in destination therapy patients with a left ventricular assist device: A qualitative content analysis
5. Krimminger and Sledge (2022) [26]	A qualitative study of life with a left ventricular assist device as a bridge to transplant: A new normal
6. Trenta et al. (2022) [27]	What is the lived experience of patients with left ventricular assist devices during the COVID‐19 pandemic? A qualitative analysis
7. Cebeci et al. (2021) [28]	A bridge to transplantation: The life experiences of patients with a left ventricular assist device
8. Neo et al. (2020) [29]	Life Beyond Heart Failure‐What Are the Long‐Term Challenges, Supportive Care Needs, and Views Toward Supportive Care of Multiethnic Asian Patients With Left Ventricular Assist Device and Their Caregivers?
9. Barg et al. (2017) [30]	LVAD‐DT: Culture of Rescue and Liminal Experience in the Treatment of Heart Failure
10. Standing et al. (2017) [31]	‘Being’ a ventricular assist device recipient: A liminal existence
11. Kitko et al. (2016) [32]	Patients' decision‐making process and expectations of a left ventricular assist device pre‐ and post‐implantation
12. Sandau et al. (2014) [33]	A conceptual definition of quality of life with a left ventricular assist device: Results from a qualitative study
13. Marcuccilli et al. (2013) [34]	Modification of self‐concept in patients with a left‐ventricular assist device: An initial exploration
14. Overgaard et al. (2012) [35]	Life in transition: A qualitative study of the illness experience and vocational adjustment of patients with left ventricular assist device
15. Casida et al. (2011) [36]	Lifestyle adjustments of adults with long‐term implantable left ventricular assist devices: a phenomenological inquiry
16. Marcuccilli et al. (2011) [37]	Sex and intimacy among patients with implantable left‐ventricular assist devices.

The quality of each included study was assessed by two reviewers (*author names removed*) using the Critical Appraisal Skills Programme (CASP) Checklist for Qualitative Research (Table 3) [38]. For reporting clarity, each study's number of ‘yes’ responses was converted into a percentage score (i.e., number of ‘yes’ items out of 10). These percentages are presented in Table 3. However, no studies were excluded based on low quality scores. No categorisation threshold was applied (e.g., high, moderate, low quality), as even studies with some methodological limitations may offer valuable insights in qualitative synthesis [18]. The CASP scores served only as a descriptive tool to assess the general rigour of included studies.

**TABLE 3:** Critical Appraisal Skills Programme (CASP) qualitative research checklist (2024).

CASP qualitative research checklist^a^											
Study	Q1	Q2	Q3	Q4	Q5	Q6	Q7	Q8	Q9	Q10	Score%
1. Bechthold et al. [22]	Y	Y	Y	Y	N	Y	Y	Y	Y	Y	90%
2. Inyom et al. [23]	Y	Y	Y	Y	Y	Y	Y	Y	C/T	Y	90%
3. Treß et al. [24]	Y	Y	Y	Y	N	Y	Y	Y	Y	Y	90%
4. Levelink et al. [25]	Y	Y	Y	Y	N	C/T	Y	N	Y	Y	70%
5. Krimminger and Sledge [26]	Y	Y	Y	Y	N	N	Y	N	Y	Y	70%
6. Trenta et al. [27]	Y	Y	Y	Y	Y	Y	Y	Y	C/T	Y	90%
7. Cebeci et al. [28]	Y	Y	Y	Y	Y	N	Y	Y	Y	Y	90%
8. Neo et al. [29]	Y	Y	N	Y	Y	N	Y	Y	C/Y	Y	80%
9. Barg et al. [30]	Y	Y	Y	Y	N	N	N	Y	Y	Y	70%
10. Standing et al. [31]	Y	Y	Y	C/T	N	N	Y	Y	Y	Y	70%
11. Kitko et al. [32]	Y	Y	Y	Y	N	N	Y	Y	Y	Y	80%
12. Sandau et al. [33]	Y	Y	Y	Y	N	Y	Y	Y	Y	Y	90%
13. Marcuccilli et al. [34]	Y	Y	Y	Y	Y	Y	Y	Y	Y	Y	100%
14. Overgaard et al. [35]	Y	Y	Y	Y	Y	N	Y	Y	N	Y	80%
15. Casida et al. [36]	Y	Y	Y	Y	Y	Y	Y	Y	Y	Y	100%
16. Marcuccilli et al. [37]	Y	Y	Y	Y	Y	Y	Y	Y	Y	Y	100%

Abbreviations: C/T = cannot tell; N = no; Y = yes.

^a^ For reporting clarity, each study's number of ‘yes’ responses was converted into a percentage score (i.e., number of ‘yes’ items out of 10). These percentages are presented in Table 3. The percentage reflects the proportion of criteria fully or partially met and provides a general sense of methodological rigour. However, no studies were excluded based on quality scores. There was not a categorisation threshold (e.g., high, moderate, low quality), as even studies with some methodological limitations may offer valuable insights in qualitative synthesis [18]. The CASP scores served as a descriptive tool to assess the general rigour of included studies.

The thematic synthesis applied Thomas and Harden's [18] methodology to analyse the qualitative data, providing a rigorous framework for understanding the complex, multifaceted experiences of LVAD patients. Through iterative coding and intersubjective validation, the study produced an in‐depth understanding of how technological, emotional and social factors intersect to shape the lived experience of LVAD recipients.

Although the approach of this review and synthesis was inductive and not initially guided by a specific theoretical framework, during data analysis it became evident that the findings aligned with key elements of the Transactional Model of Stress and Coping [39]. This model conceptualises coping as a dynamic process involving the appraisal of stressors and the deployment of strategies to manage perceived demands. It is particularly well suited to the context of chronic illness and technologically mediated care. In our study, the model provided a useful lens for interpreting how both patients and caregivers appraised and responded to the physical, emotional and existential challenges associated with LVAD implantation. This model offered a conceptual scaffold that helped the research team make sense of the evolving trajectory of the patient–caregiver experience.

### 4.3 Data Extraction and Analysis

**FIGURE 2:**
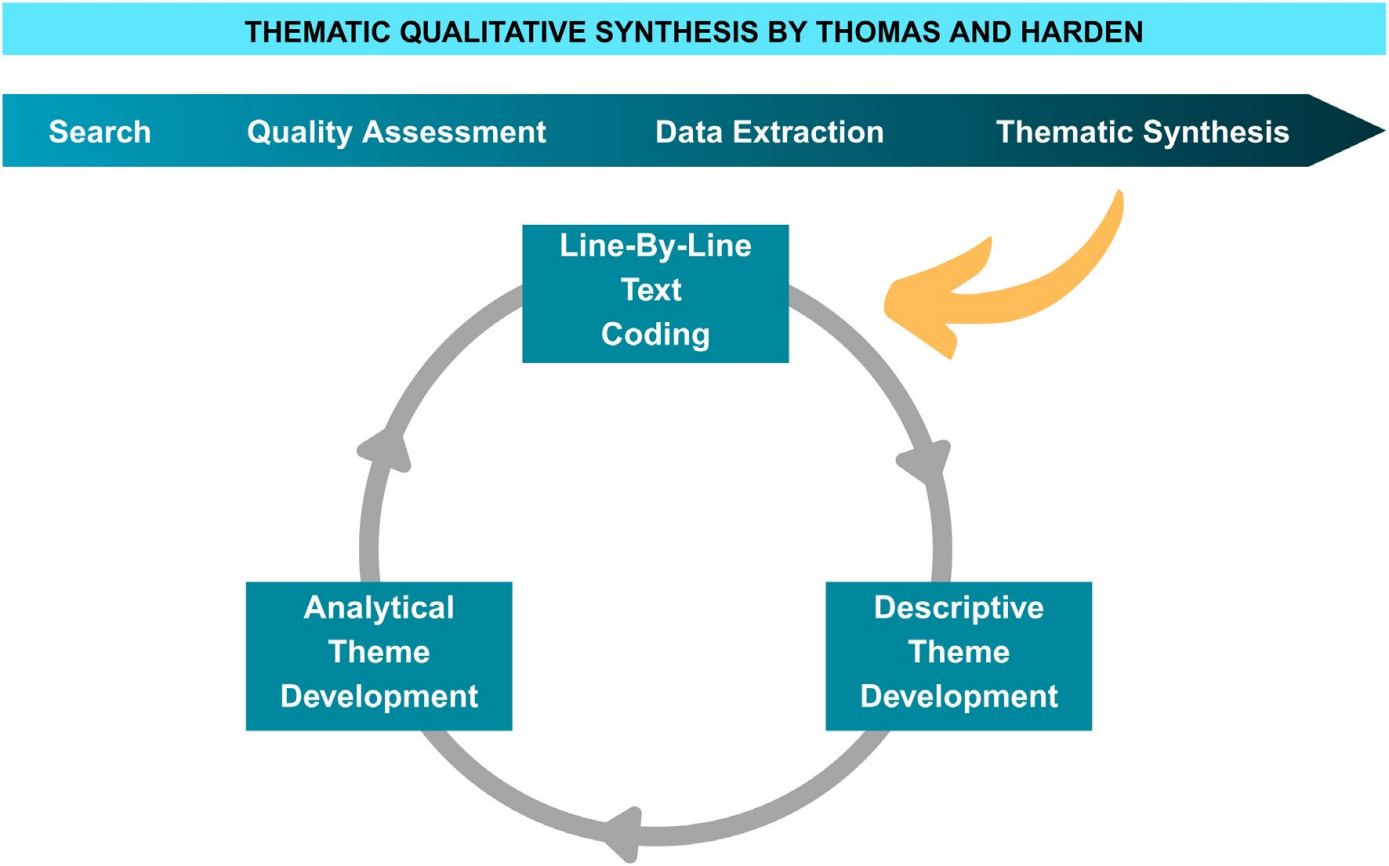
Thomas and Harden: Steps of Thematic Qualitative Synthesis. The diagram illustrates the stepwise and iterative approach used to synthesise qualitative data. The top section outlines the linear process of systematic review—Search, Quality Assessment, Data Extraction and Thematic Synthesis. The bottom cycle reflects the analytic depth of the method, showing Line‐by‐Line Coding, Descriptive Theme Development and Analytical Theme Development. This model guided the transformation of raw patient narratives into structured, theory‐informed findings.

The researchers read the articles and extracted the following information: study aim, participants, country of origin, analysis methods, strengths and limitations and main findings (Table 4). Three researchers (*author names removed*) reviewed and coded all included articles using Atlas.ti software. The synthesis followed a three‐stage process, which overlapped to some degree. The coding was performed line‐by‐line and article‐by‐article, in a back‐and‐forth manner, as recommended by Thomas and Harden [18] (Figure 2). Their thematic synthesis method is widely recognised in qualitative research for providing a structured, transparent, and rigorous approach to analysing qualitative evidence, particularly in systematic reviews [21, 40].

**TABLE 4:** Data extraction from the studies.

Study	Objective	Sample, country	LVAD type	Design	Data collection	Data analysis	Main findings	Codes and quotations
Bechthold et al. [22]	To explore the values elicitation experiences of patients with LVAD in the post‐implantation period	*N* = 27 USA Male: 16 Female: 11 Age range: 30–76 years	CF/does not mention	Qualitative descriptive study	Semi structured interviews	Data were analysed using NVivo 12 and Attride‐Stirling's Thematic analysis approach	Three themes [3] of patient values elicitation experiences emerged: (1) LVAD implantation prompts deep reflection about life and what is important, (2) patient values are communicated in various circumstances to convey personal goals and priorities to caregivers and clinicians and (3) patients leverage their values for strength and guidance in navigating life post‐LVAD implantation. LVAD implantation was an impactful experience often leading to reevaluation of patients' values; these values became instrumental to making health decisions and coping with stressors during the post‐LVAD implantation period.	Patients leverage their values for strength and guidance in navigating life post‐LVAD implantation: ‘I say it's like the three Fs, my faith, my family, my friends. When we speak of values it's like, I guess you can say like what I place on myself with those things, because those were the things that helped me through, and that continues to help me […] We joke about it now, but like my mom, she was saying how I would fight myself out of the anaesthesia […] I wanted to fight for my life, because I have kids, and I'm like, I have to be here for them’ (Female, 40‐year‐old)
Inyom et al. [23]	To describe and understand the lived experiences of patients undergoing long‐term circulatory support with LVAD	*N* = 21 Germany Male: 14 Female: 7 Age range: 37–78 years	Does not mention/CF	Qualitative Study: Phenomenological approach	Semi‐structured interviews	Colaizzi phenomenological analysis method	Patients' quality of life was affected by (1) improvements or setbacks in their recent health condition, (2) burdens from their device such as weight and handling, (3) limitations in their physical ability or reduced sleep, (4) reduced social interactions, (5) reduction in sexual activity and performance, (6) emotional and psychological problems and anxiety, (7) the presence of support from family and friends and (8) the feeling of optimism and obtaining ‘a second chance’ in life.	Limitations of LVAD device ‘The weight of this thing (pointing at the machine) is really too much for me, I wish it was a bit lighter, I have so much pain in my back and neck’ (Female, 55‐year‐old)
Treß et al. [24]	To explore the perceived health‐related needs of patients living with a LVAD in their home environment. To explore the health‐related needs of patients with LVAD in the early and late home stages	*Ν* = 10 Germany Male: 9 Female: 1 Age range: 30–75 years	Does not mention/CF	Qualitative approach‐hermeneutic, content analysis by Hsieh and Shannon	Telephone interviews	Software MAXQDA	Overarching themes [2]: (1) normalcy and (2) safety. Participants expressed a need to balance daily activities between striving for normalcy and maintaining safety. Underlying necessities reflecting this balance were categorised as functional, social and mental health‐related needs. Relevant requisites: learning by doing, social and peer support.	Learning by doing, social, and peer support: ‘So much stuff you need to carry around as soon as you are back home. You are advised as to how to handle everything, but you have to practice {…} There is no right way, you have to learn it by experience’ (Patient's demographics unavailable)
Levelink et al. [25]	To explore psychological burden, coping resources and strategies from the perspective of DT patients living with an LVAD over a time span of 3 months to more than 10 years	*N* = 18 Germany Male: 14 Female: 4 Age range: 33–78 years	CF/DT or BTT	Cross‐sectional qualitative design	Semi‐structured in‐depth interviews	Software MAXQDA‐Inductive qualitative content analysis as outlined by Elo and Kyngas	Thematic categories [7]: (1) health care issues, (2) LVAD features, (3) physical limitations, (4) comorbidity, (5) complications, (6) LVAD management and (7) health state volatility.	Burden; social aspects ‘At first, nobody talks to you. Some may have thought I was contagious. They haven't spoken to me in a while’ (Patient's demographics unavailable)
Krimminger & Sledge [26]	To explore the lived experiences of individuals with LVAD as a bridge to transplant	*N* = 10 USA Male: 9 Female: 1 Age range: 41–60 years	CF/BTT	Qualitative Descriptive Study	Semi‐structured interviews	Qualitative content analysis with an inductive coding approach	Main themes [3] encompassing several sub themes: (1) physical adjustments (reality shock, carrying the batteries, a new routine, showering and sleep), (2) emotions (decision‐making, attitude, gratitude and uncertainty) and (3) psychosocial aspects (intimacy adaptations, self‐perception and perception by others).	Reality shock ‘I feel like a bionic man. I am dependent on batteries and electric power’ (Male, 51–60 years age group)
Trenta et al. [27]	To explore the lived experience of people with LVAD during the COVID‐19 pandemic	*N* = 8 Italy Male: 8 Female: 0 Age range: 65–82 years	CF/does not mention	Qualitative Study: Interpretative phenomenological analysis	Semi‐structured in‐depth interviews—online	IPA methodology	Main themes [2]: (1) ‘Worsening of psychological distress’ and (2) ‘Moving forward’ and eight sub‐themes.	Distress for social isolation ‘What I miss is a social gathering, because I live for people, I live for social contacts. (…) A few days ago, I cried for my nephew because he was on the stairs and said “Grandpa, I'd like to hug you.” These things are heavy’ (Male, 65‐year‐old)
Cebeci et al. [28]	To examine the life experiences of patients who underwent LVAD implantation as a bridge to transplantation	*N* = 13 Turkey Male: 9 Female: 4 Age range: 28–57 years	CF/BTT	Descriptive phenomenological research	Semi‐structured in‐depth interviews	Inductive content analysis method according to Graneheim and Lundman	Themes [2]: (1) fear and (2) coping. Participants' fears and coping strategies for these were identified.	Faith ‘…God is with me, everything comes from him, what else I can ask…’ (Male, 56‐year‐old)
Neo et al. [29]	To explore the experiences of multi‐ethnic Asian LVAD patients and caregivers so as to identify their long‐term challenges, supportive care needs and views towards supportive care	*N* = 41 Singapore Patients: *N* = 30 Male: 25 Female: 3 Median age: 56 years Caregivers: *N* = 11 Male: 1 Female: 10 Median Age: 55.5 years	CF/DT BTT	Grounded theory approach	Semi‐structured interviews	Data collection, analysis and theme generation were ongoing and concurrent processes based on grounded theory	Themes [3]: (1) permanent and recurrent losses, (2) mutuality and connectedness and (3) and being holistic. These themes were presented primarily from the patients' perspective, corroborated by caregivers.	Losses on the social domains ‘Because you won't be the same ….because I cannot get pregnant right, I feel useless, as a woman I cannot give birth anymore right, the challenges changed me’ (Patient's demographics unavailable)
Barg et al. [30]	To investigate how cultural meanings associated with the LVAD inform acceptance and experience of this technology when it is used as a destination therapy. To examine whether the experience of caregivers and patients undergoing LVAD implantation was consistent with their understanding when procedure was offered	*N* = 81 USA Patients *N* = 39 Male: 32 Female: 7 from < 40 years up to > 70 years Caregivers: *N* = 42 Male: 9 Female: 33 from < 40 years up to > 70 years	CF/DT	A grounded theory approach	Open ended, semi structured interviews	Interviews analysed with NVivo 10.0	Themes [2]: (1) the powerful presence of a salvation ethos present before the implantation of the device forcing adoption of the miracle device and (2) the liminal state experienced by patients while living with the device.	Being saved ‘The only thing that I would think that should be different is that they tell you all the peaches and cream. They don't tell you the bad parts. And I never knew that until after I got the device. And I—for the last year and about eight months have been sick probably every week at that time with something or other. And lots of hospitalizations’ (Patient's demographics unavailable)
Standing et al. [31]	To uncover the experience of ‘Being’ an LVAD recipient	*N* = 31 20 patients 11 partners UK No further information on patients' demographics was included.	CF/BTT	Qualitative‐IPA (Heidegger)	Semi‐structured interviews	Interpretive phenomenology	One overarching theme: life with a VAD is a liminal existence. Subthemes [4]: (1) how the VAD imposes limitations on recipients' lives that can precipitate a loss of identity, (2) temporal disruptions, recipients' sense of time changes from authentic to inauthentic, (3) the VAD itself is liminal, it is positioned as temporary rather than as the ‘answer’ to the condition and (4) VAD recipients' projections to the future and the possibility of an end to the experience of liminality.	Temporal disruptions of the LVAD ‘It's awful, it's an awful feeling you being stuck … at this crossroads you can't go anywhere; it's like you being in a telephone box and not being able to get out’ (Patient's demographics unavailable)
Kitko et al. [32]	To examine patients' pre‐implantation decision‐making and pre‐ and post‐implantation expectations of LVADs	*N* = 15 USA Male: 11 Female: 4 Age range: 39–75 years	CF/DT‐BTT	A longitudinal qualitative design	Semi‐structured interviews, individually monthly for up to 2 years or until the patient's death	Qualitative thematic approach	Themes [3]: (1) no choice, (2) I thought I would be doing better and (3) I feel good, but now what?	Pre‐implantation: No choice ‘There is no decision when they give you the alternative. I am not ready to die’ (Patient's demographics unavailable)
Sandau et al. [33]	To develop a conceptual definition of quality of life (QoL) with LVAD.	*N* = 11 USA Male: 8 Female: 3 Age range: 38–73 years	CF/DT = 6 BTT = 4 BTR = 1	Grounded theory	Individual or paired interviews, semi‐structured	Questioning techniques for grounded theory inquiry as described by Glaser and Strauss and Krueger and Casey	A conceptual definition of QoL from patients with an LVAD: ‘being well enough to do and enjoy day‐to‐day activities that are important to me’. Five domains Physical domain: ‘There's a new normal’. Emotional domain: ‘The up and down‐ness of it’. Social domain: ‘A reason to get up in the morning’. Cognitive domain ‘It's coming. It's taking time’. Spiritual domain: ‘It gives me peace’.	Emotional domain; Anger ‘They look at it and go, “This guy is carrying a bomb.” And then they bring me over there and pat me down, telling me.to leave the [controller] bag on the table and come over here. I can't, and they don't get it. Until finally it's like, “Oh, it's connected to you. Oh, I get it.”’ (Patient's demographics unavailable)
Marcuccilli et al. [34]	To explore how patients LVAD meet the health‐deviation requisite of modifying self‐concept to accept this form of treatment and restore normalcy	*N* = 9 [same sample with [37]] USA Male: 7 Female: 2 Age Range: 31–70 years	CF/DT BTT	Hermeneutic phenomenology—van Manen	Semi‐structured interviews	Data were organised and coded into words and phrases utilising the qualitative software NVivo 8	Themes [2]: (1) Having an LVAD means living. Participants described they ‘feel alive again’ and they ‘had the rest of [their] lives that they didn't have before’ and (2) A desire to be ‘normal’ in public, arose from participants descriptions of how the LVAD brought unwanted attention to them and that their appearance was ‘shocking’ to others.	A desire for normalcy in public ‘[I was] walking into a bank you know… you see everyone's eyes are on you (laughing) and I even had a couple of guards come up to me… and grab me by the arm… [it makes me feel like] you're in a fishbowl… but again…. I've gotten used to it (being stared at in public)’ (Patient's demographics unavailable)
Overgaard et al. [35]	To explore the lived experience of patients with LVADs	*N* = 10 Denmark Male: 6 Female: 4 Age range: 23–65 years	CF/BTT	Qualitative design	Semi‐structured in‐depth interviews	Data were managed by the qualitative software NVivo 8	Themes [4]: (1) transition to illness, (2) transition to LVAD, (3) life with LVAD and (4) life after cardiac transplantation.	Vocational adjustments in the early adulthood ‘I would like to [go back to work], but I won't be able to do what I want. I don't really know what I want to do, I'm thinking whether there is a kind of education or something I could take. But I still think that I would like to return to my old job. That's why I chose that job’ (Female, 34‐year‐old)
Casida et al. [36]	To explore and describe the lifestyle adjustments made by adult recipients of a long‐term implantable LVAD	*N* = 9 [same sample with [37]] USA Male: 7 Female: 2 Age range: 31–70 years	CF/DT‐BTT	Hermeneutic phenomenology	Semi‐structured, face‐to‐face interviews	The study was guided by van Manen's method of phenomenological inquiry	One overarching theme: ‘adjustment takes time’. *Early adjustment* was highlighted by participants' concerns with *physical* (activities of daily living: sleep, hygiene, clothing, home environment) *psychological* (managing stress, developing a routine) and *environmental* aspects. *Late adjustment* was highlighted by behaviours associated with acceptance of the LVAD as an integral component of their bodies and lives. The use of coping strategies was fundamental to adapting to a new way of life. Accepting the ‘new way of living’ and appreciating the second chance at life culminated in the later stage of the adjustment process.	Early Adjustment: Changes in the Basics of Everyday Life ‘When I saw where it was going inside of me, I was like, you know, I cried at first, because I was like, all this foreign stuff in my body, you know, can it really help me live?’ (Patient's demographics unavailable)
Marcuccilli et al. [37]	To explore the experience of adults living with an LVAD, including the effect of the LVAD on their intimate and sexual functioning	*N* = 9 USA Male: 7 Female: 2 Age range: 31–70 years	CF/BTT DT	IPA	Semi structured interviews	van Manen's approach to analysis and interpretation	Three themes [3]: (1) improved sexual relations with LVAD, (2) sexual adjustment and (3) non‐sexual intimacy	Improved Sexual Relations With LVAD ‘At first, I was like, you know, making all different kind of excuses. This is when I first met him [when participant first showed the LVAD to her partner] and he was like, okay, what's the problem? And I was like, you know, I have this machine. He said it doesn't take away who you are. That's part of you. And it's been great ever since’ (Patient's demographics unavailable)

Abbreviations: BTR: bridge to recovery; BTT: bridge to transplant; CF: continuous flow; DT: destination therapy; IPA: interpretive phenomenological approach; LVAD: left ventricular assist device.

The coding process involved comparing and merging similar codes, which were then organised into 32 code categories and subsequently reduced to 7 descriptive themes. To ensure trustworthiness, all authors participated in multiple rounds of joint coding, comparison, and thematic refinement until full consensus was achieved. This collaborative approach fostered intersubjective agreement and contributed to the credibility and rigor of the synthesis. Although formal intercoder agreement statistics (e.g., kappa) were not calculated—consistent with the interpretive, constructivist orientation of our synthesis—we prioritised reflexive dialogue and consensus‐building throughout the analytic process.

The results of each study, including abstracts, quotations, authors' interpretations, tables and figures, were imported into the qualitative data management software Atlas.ti. Analytical themes were synthesised based on the descriptive themes, providing cross‐study interpretations. Differences in interpretation were discussed until consensus was reached. While descriptive themes closely align with the primary studies, analytical themes represent a deeper level of interpretation, where the reviewers go beyond the studies themselves to generate new constructs, explanations, or hypotheses [17, 41].

## 5 Findings

### 5.1 Study Characteristics

Sixteen studies (Table 2) from seven countries were included in the review, involving a total of 241 adult patients and 64 informal caregivers. Among the patients, 168 were male, 53 were female, and gender was not reported for 20 individuals. For caregivers, 34 were female, 19 were male, and 11 were of unspecified gender (this information was not reported in the respective studies). Participant ages ranged from 23 to 82 years, although three studies did not provide age data. In terms of sample size, 12 studies included between 8 and 21 participants, whereas four studies included between 27 and 81. Due to the wide variation in sample sizes, a mean value across all 16 studies was not calculated. In several of the included studies, participant demographic information (e.g., age, gender) was not consistently reported. As such, individual quotes in this synthesis may not always be accompanied by demographic details.

Table 4 summarises the key characteristics and findings of the 16 studies included in the systematic review. These studies were conducted across various countries and included diverse populations of LVAD recipients. The studies employed a range of qualitative methodologies, with common themes related to patients' physical, psychological and emotional experiences post‐implantation. The sample sizes varied, and studies explored different aspects of LVAD therapy, including adaptation, coping mechanisms, quality of life and social support systems.

### 5.2 Themes and Qualitative Synthesis

The analysis revealed four main analytical themes based on the synthesis of coded data: (1) *Loss: The Failing Body, Stigma, and the Others*, (2) *Living with Fear and Uncertainty: And Now What?* (3) *Liminality: Stagnating Present and Ambivalence* and (4) *Moving Forward and Finding New Meaning in Life* (Table 5). These themes go beyond the functional aspects of health and explore emotional, relational and lifestyle domains, focusing on how patients reconcile with their LVAD condition and strive to move forward.

1. Loss: The Failing Body, Stigma and the Others.
  This theme highlights the physical and emotional burdens associated with living with an LVAD. Patients frequently discussed the limitations posed by the device, such as its weight, the discomfort of the driveline and restrictions on daily activities like sexual function and personal appearance. As one participant said, ‘The weight of this thing (pointing at the machine) is really too much for me’ (Study 2, Female, 55 years), whereas another expressed the emotional impact, noting how the device altered their self‐image and sense of normalcy. The theme also addresses the stigma patients feel, as exemplified by one participant who mentioned, ‘I hide my wire cable… until today my boss doesn't know I'm an LVAD patient’ (Study 8), reflecting the social and psychological barriers created by the visible aspects of the device. This aligns with the theme of self‐concept, which was described in earlier studies, such as by Sandau et al. [33], who found that LVAD patients often struggled with their physical appearance and the social implications of their condition. The theme is structured into three sub‐themes: (a) body, (b) everyday challenges and (c) relationships, each reflecting critical aspects of how individuals navigate their physical and functional health.
  Firstly, the *body* reflects the physical constraints patients face, particularly with mobility limitations, chronic fatigue and functional dependency on the LVAD. The ‘failing body’ is perceived as a burden in daily life, reshaping individuals' sense of normalcy. These limitations are compounded by the constant awareness of mechanical dependency, which contributes to psychological distress, loss of autonomy and altered self‐perception. This is particularly evident in the challenges surrounding sexual and reproductive health, where patients report issues such as reduced libido, anxiety about intimacy and the loss of childbearing ability (Study 2, Study 8, Study 15). Secondly, as *everyday challenges* are described by the participants, the constant adjustments required to accommodate the LVAD in their daily routines, such as the need to carry equipment and manage the risks of device malfunction, were noted. Many reported restrictions on travel, fears about device failure and the necessity of frequent monitoring, which led to significant disruptions in lifestyle. These constraints affected personal independence and often resulted in social withdrawal. ‘So much stuff you need to carry around as soon as you are back home’ (Study 3), as one participant explained, reflecting the burden of daily life with the LVAD. These challenges were also reflected in the studies of Krimminger et al. [26] and Cebeci et al. [28]. Lastly, the *relationships* refer to the relational challenges faced by LVAD patients, as they are compounded by stigmatisation, both from society and within personal relationships. Participants reported strained relationships with family and friends due to the burden of caregiving demands, fears about the device and the need to hide the LVAD in public to avoid stigma. ‘I hide my wire cable… until today my boss doesn't know I'm an LVAD patient’ (Study 8) demonstrates how stigma creates social isolation. Decision‐making around LVAD implantation also carried emotional turmoil, as patients weighed the survival benefits against the lifestyle constraints and dependence on medical technology. As one participant in Study 11 shared, ‘There was no question about it. If I wanted to live, that was my choice I had to make’. Financial strain emerged as another challenge, with ongoing medical expenses, hospitalisations, loss of income and the costs of caregiving impacting patients' daily lives (Study 5, Study 14).
2. Living with Fear and Uncertainty: And Now What?
  This theme explores the emotional instability characterised by fear, ambiguity, uncertainty, identity disruptions and decision‐making ambivalence experienced by individuals living with LVADs. The findings reveal a complex matrix of emotional ups and downs, social stigmatisation and identity loss. Participants frequently expressed anxiety, frustration and a sense of vulnerability due to their dependence on the LVAD for survival. One participant reflected, ‘This is a machine. Machines fail. I worked with machinery for years… Anything manmade is gonna fail’ (Study 9), capturing the existential uncertainty associated with LVAD use. This uncertainty was compounded by insufficient preparation or overly optimistic expectations about survival, leading to feelings of disillusionment. Emotional instability, including anxiety and depression, was reported, as one participant shared, ‘I was depressed; like I just didn't want to talk to anybody. This was a lot to process’ (Study 12). The theme consists of two sub‐themes: (a) emotional instability and (b) fear, both of which reflect the psychological burden of living with an LVAD and the ongoing uncertainty about future health outcomes.
  Sub‐theme *emotional instability* captures all the significant emotional turbulence that patients experienced, marked by intense anxieties, depressive symptoms, altered self‐image and diminished self‐confidence. The desire for normalcy is a recurring theme, reflecting patients' longing for their pre‐illness identity. The emotional rollercoaster of hope and despair was expressed through the tension between gratitude for survival and frustration over physical limitations. One participant noted, ‘I am happy this pump has given me a second chance at life’ (Study 2, Male, 46 years), but also shared, ‘I thought when they put it in I was going to be fine and be able to do everything like I used to, well that is not the way it is’ (Study 11). The desire for normalcy, reflecting patients' longing for their pre‐illness identity, emerged as a recurring theme. These emotional struggles were compounded by a lack of holistic care, as patients navigated altered social roles and restricted activities, often leading to social withdrawal. As one participant expressed, ‘I can't get on my kayak and go down a white water river… it gets me upset’ (Study 1, Male, 45 years), highlighting how the limitations of the LVAD reshaped their social and emotional lives. The sub‐theme *fear* emerged as a persistent emotional state, encompassing both existential and situational fears. Existential fears about death or not receiving a transplant were prevalent, while situational fears related to device failure and complications were equally troubling. One participant reflected, ‘I am afraid to play with them because they like jumping around and touching several things at the same time. I am concerned that they might pull off the cable’ (Study 2). The fear of complications led to hypervigilance, sleep disturbances, and heightened anxiety, often resulting in psychological strain. Participants frequently described feeling overwhelmed by the need for constant monitoring and fear of being dependent on the device, contributing to a loss of autonomy. Furthermore, fear of stigmatisation was widespread, with patients expressing discomfort about the visibility of the device and the potential for social judgement. ‘They don't really know what it is…you kind of hide ‘em so it's not so obvious as they think you got some kind of real bad, bad issue’ (Study 5, Male, Age group: 51–60 years), one participant explained, reflecting the social avoidance and withdrawal triggered by stigma. Fear of stigmatisation was further illustrated by participants' reluctance to engage in public activities, particularly those that highlighted their condition, reinforcing isolation.
3. Liminality: Stagnating Present and Ambivalence.
  This theme explores the liminality experienced by LVAD patients, representing a state of being ‘in‐between’ life and death, health and illness, normalcy and abnormality. Many patients described their condition as a ‘borderline’ existence, where they felt neither fully sick nor fully healthy, echoing findings by Standing et al. [31]. This experience of liminality was characterised by ambivalence, as patients could not fully embrace their ‘new normal’. One participant reflected, ‘It's awful, it's an awful feeling you being stuck… at this crossroads you can't go anywhere’ (Study 10), capturing the psychological stagnation many felt. Others described their lives as suspended, noting, ‘Life is normal about half of the time’ (Study 14, Male, 47 years), which highlights the uncertainty and temporality of their existence. This theme is structured around the sub‐themes of (a) liminality and temporal disruptions and (b) loss of identity. Patients experienced a stagnating present, where their lives were sustained but not fully lived, leading to existential crisis and ambivalence. As one participant noted, ‘Provided it [heart transplant] is going to happen eventually you just keep on day‐to‐day with just getting on with life … it's [the VAD] keeping me alive until such time as I can get the heart transplant’ (Study 10), exemplifying the feeling of being trapped in a prolonged state of uncertainty.
  Firstly, the sub‐theme of *liminality and temporal disruptions* captures the psychological and social ambiguity that LVAD patients face, torn between their pre‐illness identity and their new, medicalised self. Many patients felt that they were neither truly alive nor fully ill, leading to emotional instability and a profound sense of disconnection from both healthy individuals and terminally ill patients. One participant remarked, ‘My life was on standby until the heart surgery…’ (Study 14, Male, 24), underscoring the sense of time being distorted by the LVAD experience. The sense of temporal disruption was a common theme across studies, where participants felt ‘stuck’ in an indefinite state, as described by a participant who said, ‘It's swings and roundabouts’ (Study 10), symbolising the unpredictable nature of their daily existence. This existential uncertainty often led to social isolation and fragmented identity, as individuals struggled to reconcile their former self with the reality of living with a life‐sustaining device. Secondly, the *loss of identity* refers to the sense of being ‘forever connected to a machine’ was particularly impactful in terms of self‐perception. Patients frequently discussed how the device redefined their sense of self and the roles they once held. As one participant expressed, ‘I take great pride in my ability to fulfill my responsibilities as a father… now, I have to figure out how I can do it, or can I do it at all’ (Study 12), highlighting the loss of identity tied to the LVAD. This sense of identity loss was often accompanied by a struggle for meaning, as patients questioned their purpose and place in the world after receiving the LVAD. As noted by participants, the device became a constant reminder of their vulnerability and mortality, reshaping their relationships and how they engaged with the world around them.
4. Moving Forward and Finding Purpose in Life
  This theme captures how LVAD recipients move forward despite significant challenges, seeking to rebuild their lives, find purpose and adapt to a new normal. Despite the hardships, many participants found new meaning in life after implantation, including the development of new social roles, the pursuit of meaningful activities and the adoption of self‐care routines. Emotional reactivity, such as overcoming anger, depression and anxiety, played a central role in this adaptation process. The concept of self‐care specifically highlights how regaining autonomy and developing self‐management skills significantly contributed to improved quality of life. In line with the findings by Levelink and Brütt [2], positive coping strategies, such as comparing oneself with others who had similar experiences, were essential for psychological adaptation. One participant shared, ‘I'm happy this pump has given me a second chance at life’ (Study 2), expressing gratitude for the opportunity to continue living. Patients also described significant lifestyle changes that reflected their newfound autonomy. As one participant noted, ‘Now, I cook what I've never done before. And I do it all, including buying groceries’ (Study 4), illustrating the development of personal control over daily activities, which contributed to a sense of purpose and empowerment. This theme consists of two sub‐themes: (a) Problem‐Focused Solutions and (b) Emotion‐Focused Approach.
  The sub‐theme of *problem‐focused solutions* reflects how LVAD patients adopt practical, proactive solutions to regain control over their lives. These adjustments include managing the technological complexities of the LVAD, such as monitoring battery life and troubleshooting device issues, as well as modifying their home environments to ensure safety and comfort. One participant shared, ‘Now, I don't even look at that [driveline]. I don't even pay attention to the driveline with the lines going to my stomach. I don't pay attention to that. The whole thing about it is feeling better; going to bed with this equipment, getting up with this equipment, um, is living’ (Study 15). Patients also emphasised adopting healthier habits, such as quitting smoking, to maintain their well‐being. These adaptive strategies restored functional independence and improved health, but they required continuous problem‐solving and resilience. Sexual and reproductive health challenges were addressed through open communication with partners and healthcare providers as well as adjusting expectations and redefining intimacy. One participant noted, ‘I guess that, at this point, [sex] hasn't been maybe that important in my life right now. But just being able to snuggle up in bed sitting next to each other on the sofa, even things like that are really important to me now’ (Study 16, Female, 70). In addition, many patients engaged in activities to enhance social integration, which helped combat social isolation and stigma, and fostered meaningful relationships. On the other hand, the sub‐theme of the *emotion‐focused approach* centres on emotional transformation, acceptance and the development of a positive outlook on life. Patients exhibited emotional resilience, cultivating hope and optimism as part of their coping strategies. Viewing the LVAD as a second chance at life was a common perspective, which helped patients reframe their experiences positively and envision a better future. As one participant expressed, ‘If they keep going with this machinery I'll be around for a long time’ (Study 9). Spirituality also played a crucial role in fostering a positive outlook. A few patients engage in spiritual practices to cope with uncertainties, gain emotional strength and cultivate gratitude for prolonged life. A participant noted, ‘I believe in a Higher Power and the strength from that. Those around you where you can find your strength’ (Study 12). Faith in God and trust in the LVAD itself were seen as sources of existential comfort and meaning. One participant noted, ‘To wake up every morning and see a new day and learn to accept life and be grateful. It means so much to me now where I took it for granted before I got my LVAD. I didn't think much about waking up. Every day now I wake up and I'm grateful and I'm thankful’ (Study 1, Female, 53 years). Many patients described an emotional journey from fear and despair to acceptance, gratitude and even humour, which helped them navigate their new reality with greater ease.

**TABLE 5:** Data analysis: descriptive and analytical themes.

	Theory phases	Analytical themes (4) and subthemes	Descriptive themes (7)	Code categories (32)
Transactional model of stress and coping	Primary appraisal	Loss: the failing body, stigma and the others — Body (A) — Everyday challenges (A, B, D, F) — Relations (B, D)	Physical and Functional Dimensions of Life	A.1. Physical Limitations and Challenges A.2. Everyday Life Activities A.3. Sexual and Reproductive Health A.4. Overall Health and Well‐being A.5. LVAD characteristics and Limitations
	Secondary appraisal	2 Living with fear and uncertainty: And now what? — Emotional instability (A, B, D, F) — Fear (B)	B Emotional—Psychological Dimensions	B.1. Anxiety and Emotional Problems B.2. Lifestyle Changes and Social Aspects B.3. Attitude and Decision Making B.4. Fear and Concerns B.5. Loss and Search for Meaning
		3 Liminality: Stagnating present and ambivalence — Liminality/temporal disruptions (B, G) — Loss of identity (G)	C Hope and Optimism	C.1. Second Chance at Life C.2. Acceptance and Hope C.3. Possibility for a Better Future
	Problem and emotion‐focused solving strategies	4 Moving forward and finding purpose in life — Problem‐focused solutions (A, B, E) — Emotion‐focused approach (B, C, D, E, F)	D Social Aspects and Relations to Others	D.1. Reduced Social Interactions D.2. Maintaning Relationships D.3. Perception and Stigmatisation D.4. Financial Strain D.5. Connectedness D.6. Desire for Normalcy
			E Adaptation and Coping Mechanisms	E.1. Health‐related Balance E.2. Coping Strategies E.3. Moving Forward E.4. Spirituality E.5. Adjustment over time
			F Receiving Support from Others as part of Holistic Care	F.1. Asking for Support F.2. Importance of Social Support from Family and Friends F.3. Need of Holistic Care from Healthcare Professionals
			G Liminality and Ambivalence	G.1. Salvation Ethos and Adoption of the Device G.2. Liminal State G.3. Loss of Identity G.4. Temporal Disruptions G.5. Liminality of the VAD

## 6 Discussion

This review and synthesis reveal the intricate emotional, social and existential challenges faced by individuals living with LVAD, many of which have roots in their critical care trajectory. The findings offer a rich narrative of the patient's journey, which often begins in the intensive care unit (ICU) following acute decompensation or surgical implantation of the device. The psychological, emotional and physical transitions that follow extend into the post‐discharge continuum. The themes that emerged from our analysis—Loss, Emotional Instability and Ambivalence, Liminality and Moving Forward—highlight a gradual transformation from bodily vulnerability to psychosocial resilience, a process highly relevant to ICU nurses tasked with supporting patients during the earliest, most critical phases of this journey. These findings align with existing literature, confirming the multidimensional nature of living with an LVAD [42, 43, 44].

**FIGURE 3:**
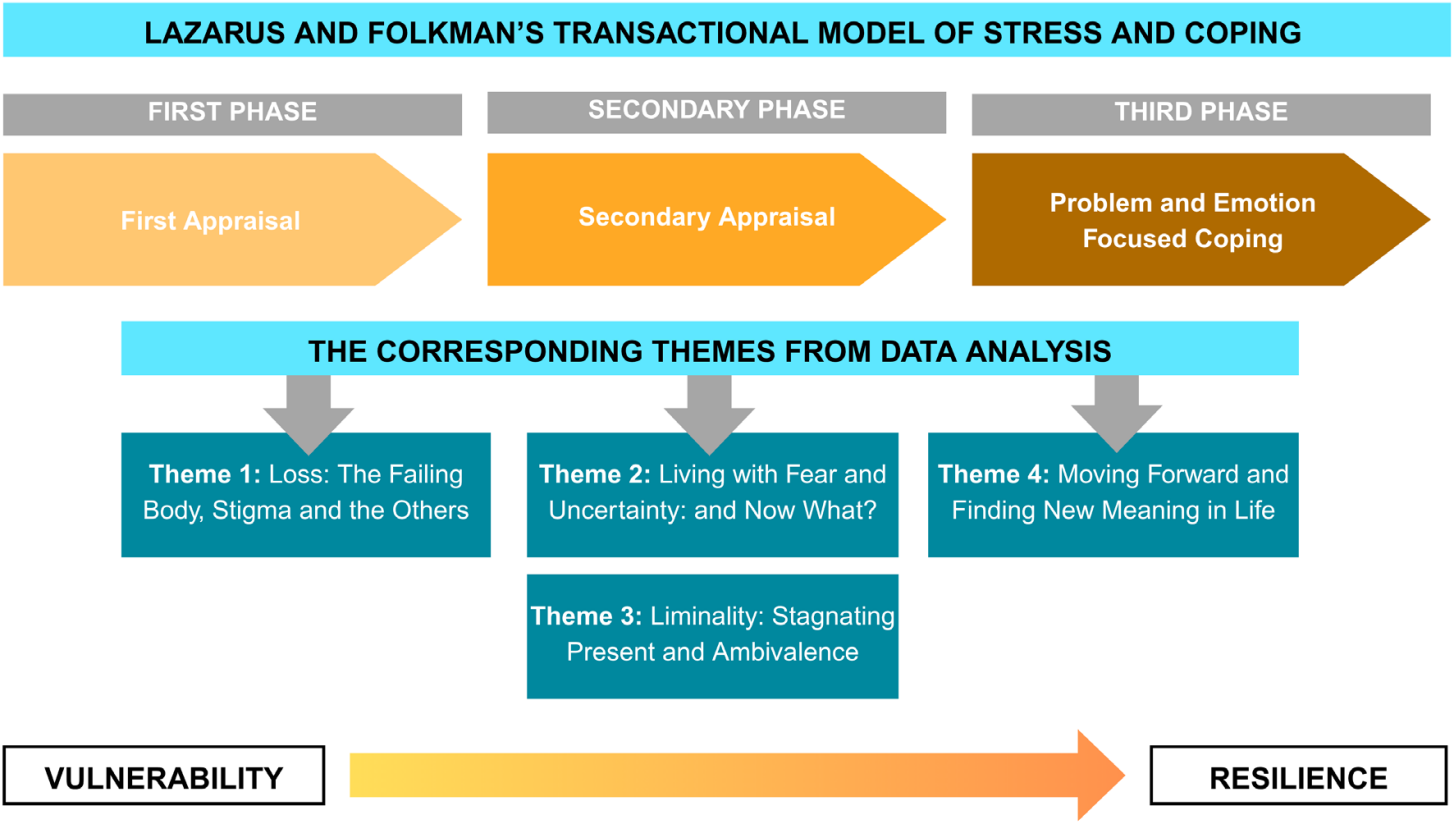
Transactional model of stress and coping by Lazarus and Folkman and the corresponding themes emerging from this meta‐synthesis. The diagram illustrates how the participants' lived experiences align with the model's core phases: Primary Appraisal, Secondary Appraisal and Coping strategies. Emergent themes—including perceptions of loss, uncertainty, emotional adjustment and adaptation—are mapped onto this framework to highlight the dynamic process of stress evaluation and coping over time. The conceptual layering reflects the progression from vulnerability to resilience across the LVAD journey.

These findings align with the Transactional Model of Stress and Coping [39]. This model provides a valuable framework for understanding how LVAD patients navigate the emotional, social and psychological challenges of their condition. The model's components—primary and secondary appraisal, problem‐focused coping and emotion‐focused coping—explain how patients manage their complex experiences. Incorporating these concepts into clinical practice can help healthcare providers address both the medical and the emotional needs of LVAD patients, improving their quality of life and fostering a sense of control, resilience and purpose. Patients' and caregivers' narratives reflected ongoing cognitive appraisals, often shifting between threat, challenge and loss. The ICU‐to‐home transition emerged as a particularly acute context for such coping demands, reinforcing the model's relevance for understanding long‐term adaptation. The authors mapped the analytical themes onto the model's three core components: Theme 1 (Loss) aligned with *Primary Appraisal*, Themes 2 and 3 (Emotional Instability and Liminality) with *Secondary Appraisal* and Theme 4 (Moving Forward) with *Problem‐and Emotion‐Focused Coping* strategies (Figure 3).

This theoretical framework helps frame the patient's journey from initial distress to psychological adaptation. The concept of *Primary Appraisal* helps frame the patient's recognition of bodily decline, technological dependence and stigma as threats to selfhood and independence [42, 44]. These threats are not abstract but materialise during the ICU stay, where patients undergo invasive procedures, loss of bodily control and sometimes sedation‐related delirium, all of which alters their sense of self [45]. ICU‐acquired weakness and cognitive changes can amplify feelings of loss and foster early identity disruption, contributing to the emotional instability seen in our findings [22, 46, 47, 48]. The liminality experienced by patients—feeling suspended between life and death, health and illness—further exemplifies how stressful factors are continuously reappraised, often leading to emotional instability and a diminished sense of normalcy [46, 48]. Additionally, the social stigma associated with visible bodily changes, such as the LVAD device, leads to social isolation and emotional distress.

In the *Secondary Appraisal* phase, patients evaluate their coping resources and begin to process their emotional instability and ambivalence. Existential fear, emotional turbulence and moral dilemmas surrounding implantation decisions were widely reported [48, 49]. As in the ICU context, LVAD patients often emerge from critical illness into prolonged uncertainty, experiencing post‐ICU syndrome, anxiety and depression [47, 50]. Harris et al. [43] also noted that body and mind integration issues during cardiac rehabilitation contribute to emotional distress and identity challenges. The emotional shifts observed, including anxiety, depression and fear of device failure or death, are part of the primary appraisal, but they are also reappraised in the secondary phase as patients seek out resources to manage these emotions. These emotional states are compounded by the liminal experience of being suspended between life and death—a condition that is psychologically taxing and socially isolating. Critical care nurses play a vital role during this phase by offering emotional reassurance and initiating structured interventions to prepare patients for life beyond the ICU [51]. Additionally, spiritual practices and reframing the LVAD experience as a second chance at life help patients manage their emotional instability. This reflects emotion‐focused coping strategies, where patients try to reinterpret the emotional toll of their condition in a more manageable way [48, 49]. It is worth noting that ambivalence also plays a central role in secondary appraisal. The decision to accept or reject the LVAD, or to face the possibility of not receiving a heart transplant, creates moral and existential dilemmas. These conflicting emotions—gratitude for survival mixed with frustration over physical limitations—highlight the ambivalence experienced by patients in this phase. The secondary appraisal leads to a complex mix of emotions, where patients re‐evaluate their coping resources and adjust to their new reality [42, 48].

The third phase involves *Active Coping*. Regaining Control is central to this phase. In this study, patients developed problem‐focused strategies such as adhering to complex medical routines, modifying their environments and engaging in health‐promoting behaviours [52, 53]. Emotion‐focused coping was also prevalent, particularly through spiritual practices and reframing the LVAD experience as a second chance at life. The acceptance of the LVAD as a second chance at life reflects the cognitive reappraisal process, where stressors are reinterpreted in a more manageable and meaningful way. Most importantly, social support—from caregivers, families and professionals—emerged as a buffering resource. Within critical care, this highlights the role of ICU nurses not only in technological management but also in shaping long‐term coping capacity. However, social avoidance and withdrawal due to fear of stigmatisation were also reported by patients, with some choosing to hide the device from others. This social withdrawal can be seen as a coping mechanism where patients attempt to regain control over how they are perceived in social settings, thus reducing the psychological burden of stigma. As mentioned by Gillilan et al. [46] and Hallas et al. [54], this behaviour reflects an effort to maintain control over personal identity and social roles. ICU follow‐up clinics, nurse‐led discharge protocols and structured transitions to outpatient care are all recommended practices that could reinforce such coping strategies [55, 56].

It is worth noting that the Transactional Model of Stress and Coping by Lazarus and Folkman [39] continues to be widely applied in contemporary nursing research, offering a robust framework for exploring stress appraisal and coping mechanisms in both clinical and educational settings [57, 58, 59]. Its sustained relevance highlights its value in examining the emotional and psychological responses of patients, nurses and students within complex healthcare environments. These findings also resonate with Boss's theory of ambiguous loss, which conceptualises prolonged uncertainty and identity fragmentation as a form of unresolved grief experienced in ongoing, ambiguous situations [60]. While the Transactional Model of Stress and Coping helps frame patients' appraisal and coping strategies, Boss's framework enriches our understanding of the emotional ambivalence and suspended identity experienced by LVAD recipients, especially in their prolonged dependence on a life‐sustaining device without a clear resolution.

The findings underscore the central role of interpersonal relationships and social support in the recovery process for LVAD patients. Emotional encouragement from family, friends and healthcare professionals fosters resilience and enhances emotional stability, while mutual support between patients and caregivers helps to alleviate the burdens associated with LVAD dependence. Our synthesis highlights that emotional and existential recovery often begins at the bedside: early conversations, spiritual support and psychological interventions initiated during critical illness can help to mitigate distress and facilitate adaptation [47, 56]. Preparing LVAD patients for discharge also requires addressing their vulnerabilities—fear, stigma, altered self‐image and sexual health concerns—within a person‐centred and holistic framework [48].

Throughout the LVAD trajectory, holistic care approaches that incorporate psychological counselling, robust social support systems and spiritual care were consistently identified as essential [48, 56]. Patients who embraced optimism and sustained hope for survival appeared better equipped to navigate complex decisions, develop proactive coping strategies and engage meaningfully in rehabilitation [51, 60]. Many participants expressed renewed hope and gratitude, describing life post‐LVAD as a second chance and found meaning by supporting others facing similar experiences—an act that helped counter feelings of stagnation and helplessness [8, 47, 61]. These studies underline the emotional, psychological and practical challenges faced by both patients and caregivers during the initial adjustment to LVAD. The study calls for healthcare providers to address these challenges through tailored psychological support and counselling.

ICU nurses involved in LVAD management should also be trained to recognise the psychosocial dimensions of care. A recent study by El Zein et al. [8] emphasised the importance of nurses' perceptions and educational needs in caring for LVAD recipients, pointing to a clear gap in confidence when addressing emotional and existential distress. By adopting the Transactional Model of Stress and Coping, nurses can better understand how to support patients through assessment, emotional support and individualised coping plans. ICU nurses are uniquely positioned to initiate anticipatory guidance, support early meaning‐making and prepare patients for the demands of LVAD life beyond hospital discharge [8, 51]. More specifically, the systematic review by Cuzco et al. [51] explored the role of ICU nurses in empowering patients during the critical transition from the ICU to general wards and highlighted the importance of nursing interventions in addressing the emotional, physical and psychological challenges faced by ICU survivors, particularly during discharge. Empowerment strategies, such as providing tailored information, education and psychological support, are emphasised as key tools to improve patient outcomes and satisfaction. ICU nurses can adapt empowerment strategies to address the unique needs of LVAD patients, though a range of interventions, such as education on device management, psychological support, collaborative care planning and follow‐up support.

Our synthesis reinforces calls for robust ICU follow‐up programs [55] and supports equipping ICU nurses with tools to better understand and intervene in the psychosocial aftermath of advanced therapies like LVAD. The relevance of this review lies in how it bridges acute care with long‐term survivorship—highlighting critical care nurses' responsibility in laying the foundation for resilience, dignity and coping far beyond the ICU walls.

## 7 Limitations

This review has several limitations. First, the inclusion of only English‐language studies may have introduced language bias. Additionally, the review focuses on qualitative studies, which may limit generalisability. The studies included in this synthesis were conducted in specific regions and settings, reflecting the geographic distribution of available qualitative research on LVAD patient experiences. Finally, the heterogeneity of the included studies, in terms of study design and patient characteristics, could affect comparability. Despite these limitations, the strength of this review lies in its methodological rigour. A transparent, systematic approach was used, including a comprehensive search strategy and critical appraisal. The thematic synthesis methodology provides a nuanced understanding of LVAD patients' experiences, ensuring that findings are grounded in data while maintaining analytical depth.

## 8 Implications for Practice

This synthesis highlights the critical role ICU nurses play in shaping the emotional, psychological and existential recovery trajectories of LVAD patients, beginning from the acute care phase through post‐ICU survivorship. ICU care often marks the start of patients' experiences of loss, fear and identity disruption, themes that are not confined to the hospital stay but extend into long‐term recovery. Evidence suggests that emotional instability and liminality, common in LVAD patients, are intensified during ICU care and require structured nursing interventions aimed at psychological support and patient empowerment [51, 56]. Furthermore, ICU nurses are in a unique position to facilitate coping strategies through early identification of psychological distress and fostering trust, emotional safety and continuity of care [45, 50]. The lack of LVAD‐specific training among ICU nurses, as identified by El Zein et al. [8] in their study in Lebanon, underscores the need for targeted education and the development of protocols that support holistic care, including preparation for post‐ICU life. ICU follow‐up clinics, which have been shown to improve critical care survivors' outcomes [47], can be leveraged to address ongoing emotional challenges such as fear of device failure and social reintegration. Nurses should also engage in values‐based care, helping patients find meaning and reconstruct identity after ICU discharge [22]. This highlights the importance of holistic, patient‐centred approaches in critical care nursing, integrating psychosocial support, empowerment and long‐term follow‐up to improve outcomes for LVAD patients transitioning from ICU to community life.

## 9 Conclusion

This study offers a comprehensive perspective on the lived experiences of LVAD patients, revealing that LVAD dependency is a biopsychosocial challenge shaping individuals' lives. We explore the liminal experience, including psychological and existential dimensions such as temporal disruption, identity fragmentation and ambivalence. The alignment with existing studies reinforces the credibility of our findings, contributing new insights into the interplay between medical dependency and identity crisis. The emotional journey of LVAD recipients demonstrates a shift from despair and instability to hope, peace and gratitude, suggesting existential transformation. The transition to ‘living with LVAD’ is not only individual but also a shared experience with family and caregivers, where trust in nurses and doctors and reliance on a care support service are crucial for recovery and motivation. Our findings highlight the importance of problem‐focused coping, emotional resilience, social support and holistic care models. The resourcefulness of patients in regaining control underscores the need for improved clinical practices. This analysis advocates for comprehensive care strategies that address physical, emotional, social and relational dimensions, promoting patient‐centred, stigma‐sensitive healthcare. The exploration of underrepresented issues, such as sexual health and intimate relationships, points to areas needing further research.

## Author Contributions

Conceptualization: D.K. and S.P. Methodology: S.P. and D.K. Supervision: S.P. Validation: A.K. and E.M. Data analysis: D.K., E.B.S. and S.P. Writing – original draft preparation: D.K. Writing – review and editing: D.K., S.P., A.K., E.M. and F.T. visualization: E.B.S. and S.P. All authors have read and agreed to the published version of the manuscript.

## Funding

The authors have nothing to report.

## Ethics Statement

The authors have nothing to report.

## Consent

The authors have nothing to report.

## Conflicts of Interest

The authors declare no conflicts of interest.

## Supporting information


**Table S1:** NICC70295‐sup‐0001‐Supinfo.docx

## Data Availability

The data that support the findings of this study are available from the corresponding author upon reasonable request.
