# Supplementary material for: A Thematic Qualitative Synthesis on the Lived Experiences of Patients With Left Ventricular Assist Devices: A Journey From Vulnerability to Resilience
Source: Nurs Crit Care. 2026 Jan 9;31(1):e70295. doi: 10.1111/nicc.70295 (PMC12784241; doi:10.1111/nicc.70295)
Supplement: Supplementary file 1 — Table S1: NICC70295‐sup‐0001‐Supinfo.docx [file NICC-31-0-s001.docx]

# ENTREQ Checklist – Completed

**Type of paper**: review article, thematic qualitative synthesis

**Title**: A Thematic Qualitative Synthesis on the Lived Experiences of Patients with Left Ventricular Assist Devices: A Journey from Vulnerability to Resilience

| **Item** | **Guide and Description** | **Reported on Page #** |
| --- | --- | --- |
| Aim | State the research question the synthesis addresses | Title & page 1, 3  Table 1 at page 3  Our aims section clearly states the objectives of synthesizing the lived experiences of LVAD patients and analyzing them through the lens of the Transactional Model of Stress and Coping. |
| Synthesis methodology | Identify the synthesis methodology or theoretical framework which underpins the synthesis, and describe the rationale for choice of methodology (e.g. meta-ethnography, thematic synthesis, critical interpretive synthesis, grounded theory synthesis, realist synthesis, meta-aggregation, meta-study, framework synthesis). | page 1, 3, 5  Figure 2 at page 6 |
| Approach to searching | Indicate whether the search was pre-planned (comprehensive search strategies to seek all available studies) or iterative (to seek all available concepts until theoretical saturation is achieved). | page 1, 3, 4  Figure 1 PRISMA at page 4 |
| Inclusion criteria | Specify the inclusion/exclusion criteria (e.g. in terms of population, language, year limits, type of publication, study type). | page 4  Figure 1 PRISMA at page 4  Bulleted list with inclusion criteria for the studies of this review |
| Data sources | Describe the information sources used (e.g. electronic databases, grey literature databases, organisational websites, experts, generic web searches, hand searching, reference lists) and when the searches were conducted; provide the rationale. | page 1, 3  Figure 1 PRISMA at page 4 |
| Electronic Search strategy | Describe the literature search (e.g. provide electronic search strategies with population terms, clinical or health topic terms, experiential or social phenomena related terms, filters for qualitative research and search limits). | page 3, 4  Figure 1 PRISMA at page 4  At **PubMed** the search strategy is presented at page 4 of the main text.  At **Science Direct** and **Scopus** search strategy:  (Lvad OR "left assist device") AND ("patient experience" OR "experience" OR "experiences") AND ("qualitative research" OR "phenomenology" OR "grounded theory" OR "thematic analysis")  At **Scopus** search strategy:  TITLE-ABS-KEY((lvad OR "left ventricular assist device") AND ("patient experience" OR experience OR experiences) AND ("qualitative research" OR phenomenology OR "grounded theory" OR "thematic analysis"))  AND (DOCTYPE(ar) OR DOCTYPE(re)) |
| Study screening methods | Describe the process of study screening and sifting (e.g. title, abstract and full text review, number of independent reviewers who screened studies) | page 4, 5  Yes, the Methods section describes the two-stage screening process, including manual, double-blind screening and full-text review, with a flowchart provided. |
| Study characteristics | Present the characteristics of the included studies (e.g. year, country, population, data collection, methodology, analysis, research questions). | page 6, Table 4 at page 6 |
| Study selection results | Identify number of studies screened and reasons for exclusion, preferably in a figure/flowchart. | page 4, 5  Figure 1 PRISMA at page 4 |
| Rationale for appraisal | Describe the rationale and approach used to appraise the included studies or selected findings. | page 4, 5  Transparent approach  Yes, the Methods section describes the two-stage screening process, including manual, double-blind screening and full-text review, with a flowchart provided. |
| Appraisal items | State tools, frameworks and criteria used to appraise the studies or findings. | page 4, 5  Table 3 CASP at page 5  the Methods section mentions the use of the CASP criteria for quality assessment. |
| Appraisal process | Indicate if the appraisal was done independently by more than one reviewer and if consensus was required. | page 4, 5  Yes, the Methods section mentions the use of the CASP criteria for quality assessment by two reviewers |
| Appraisal results | Present results of quality assessment and which articles were weighted/excluded based on this. | page 4, 5  Table 2 studies included at page 5  Table 4 data extraction at page 6 |
| Data extraction | Indicate which sections were analyzed and how data were extracted. | page 5, 6 |
| Software | State the computer software used. | page 4 covidence  page 6 atlas.ti |
| Number of reviewers | Identify who was involved in coding and analysis. | page 5, 6 |
| Coding | Describe the coding process. | page 6 |
| Study comparison | Describe how comparisons were made within/across studies. | page 5, 6  Table 5 at page 7  Iterative coding, intersubjective validation, Thomas and Harden methodology, we developed descriptive and analytical themes |
| Derivation of themes | Explain if themes were derived inductively or deductively. | page 6, 7  inductively  Table 5 themes at page 7  Figure 2 steps of methodology at page 6 |
| Quotations | Provide participant quotations and distinguish from author interpretations. | page 7 at table 5  pages 7-11 results |
| Synthesis output | Present compelling results beyond summary: interpretations, models, concepts. | page 11-16  Discussion  Conclusion  Implications for practice  Figure 3 Transactional model of stress and coping at page 12 |
